# A comparative study of broccoli sprouts powder and standard triple therapy on cardiovascular risk factors following *H.pylori* eradication: a randomized clinical trial in patients with type 2 diabetes

**DOI:** 10.1186/2251-6581-13-64

**Published:** 2014-05-28

**Authors:** Parvin Mirmiran, Zahra Bahadoran, Mahdieh Golzarand, Homayoun Zojaji, Fereidoun Azizi

**Affiliations:** 1 Nutrition and Endocrine Research Center, and Obesity Research Center, Research Institute for Endocrine Sciences, Shahid Beheshti University of Medical Sciences, Tehran, Iran; 2 Department of Clinical Nutrition and Diet Therapy, Faculty of Nutrition Sciences and Food Technology, National Nutrition and Food Technology Research Institute, Shahid Beheshti University of Medical Sciences, Tehran, Iran; 3 Research Center for Gastroenterology and Liver Disease, Department of Gastroenterology and Liver Diseases, Shahid Beheshti University of Medical Sciences, Tehran, Iran; 4 Endocrine Research Center, Research Institute for Endocrine Sciences, Shahid Beheshti University of Medical Sciences, P.O.Box: 19395–4763, Tehran, Iran

**Keywords:** Helicobacter pylori, Standard triple therapy, Broccoli sprouts powder, High-sensitive C reactive protein, Cardiovascular risk factor

## Abstract

**Background:**

In this clinical trial we compared the effects of broccoli sprouts powder, as an alternative and complementary treatment, to those of standard triple therapy, as a common medical treatment, on cardiovascular risk factors following the *H.pylori* eradication in patients with type 2 diabetes.

**Methods:**

Eighty-six type 2 diabetic patients with positive *H.pylori* stool antigen test (HpSAg) were randomized to receive one of the three following regimens: STT) Standard triple therapy (omeprazole 20 mg, clarithromycin 500 mg, amoxicillin 1000 mg, twice a day for 14 days), BSP) 6 g/d broccoli sprouts powder for 28 days, and combination of these as STT + BSP. After 4 weeks of treatment, *H.pylori* eradication rates were assessed by HpSAg. Anthropometric measures, blood pressure, serum lipids and lipoproteins as well as serum high sensitive- C reactive protein were also assessed at baseline and at the second examination.

**Results:**

Seventy-seven participants completed the study [STT (n = 28), BSP (n = 25), STT + BSP (n = 24)]. The *H.pylori* eradication rates were 89.3%, 56.0% and 91.7% in STT, BSP and STT + BSP groups, respectively. After the treatment, both systolic and diastolic blood pressure significantly decreased in STT + BSP group (*P < 0.05*). Serum triglycerides and TG/HDL-C ratio increased in STT patients group (*<0.05*). Serum hs-CRP levels significantly decreased in the patients who were treated with BSP per se (3.0 ± 2.5 at baseline *vs.* 2.3 ± 2.1 after the treatment, *P < 0.05*).

**Conclusion:**

Compared to standard triple therapy, BSP regimen in addition to considerable effects on *H.pylori* eradication had also favorable properties on cardiovascular risk factors following the *H.pylori* eradication.

## Introduction

Current data suggest that patients with type 2 diabetes are more prone to some infectious diseases including *helicobacter pylori* infection, with a higher prevalence of this infection has been reported in diabetes patients as compared to non-diabetes [1,2]. In fact, there is a bilateral relation between *H.pylori* infection and type 2 diabetes and this infection is proposed as a major contributing factor for development of insulin resistance, sub-clinical inflammation, long-term diabetes complications and cardiovascular risk factors [3-5]. Patients with diabetes are also more resistant to common treatments of *H.pylori* infection and the risk of re-infection in these patients is higher in the non-diabetes subjects [3,6]. On the other hand, since undesirable side effects may occur during treatment with common anti *H.pylori* regimens which could affect diabetes management it is more important to choose the ideal treatment for optimal *H.pylori* eradication, with high efficacy, and low risk of recurrent and side effects in patients with type 2 diabetes compared to others. The use of natural antibacterial agents including phytochemicals and bioactive nutraceuticals for *H.pylori* eradication is currently considered as a new approach with more effectiveness and safer outcomes [7-9]. Broccoli sprouts due to its high content of sulforaphane (SFN), a most potent bactericidal agent, is recently being considered for treatment of *H.pylori* infection and related disorders [10-12].

In the current study we aimed to compare the effects of broccoli sprouts powder, as both alternative and complementary treatments, with standard triple therapy (omeprazole + clarithromycin + amoxicillin), as a common medical treatment, on cardiovascular risk factors following the *H.pylori* eradication in patients with type 2 diabetes.

## Methods

### Study population

In this randomized clinical trial men and women, aged 25–60 years, with a clinical diagnosis of type 2 diabetes for at least one year, who referred to Iran Diabetes Society and endocrinology clinic of Taleghani Medical Center were recruited. The purpose and protocol of the study were explained for the patients. Exclusion criteria were including: previous treatment of *H.pylori*, consumption of PPIs, H_2_ receptor antagonists, bismuth compounds or antibiotics during the last month, previous gastric surgery, severe illness, gestation or lactation, estrogen therapy, take of vitamin K-antagonists or antioxidant supplements. Two hundred eighty-four patients with type 2 diabetes were eligible to participants in the study, of those 93 patients refused to participants and 191 patients were assessed for *H.pylori* infection.

Ethical committee of the Research Institute for Endocrine Sciences of the Shahid Beheshti University of Medical Sciences approved this clinical trial and informed written consents were obtained from all participants. The trial was registered with the following identification: IRCT 201111081640N6. The results are reported according to Consolidated Standards of Reporting Trials guidelines 2010.

### H.pylori detection, randomization, and intervention

To detect *H.pylori* infection, stool samples were obtained from all participants. Then samples were tested by stool antigen test (HpSAg) ELISA kits, according to the manufacturers' instructions. Participants were considered as positive *H.pylori* infection if HpSAg ≥ 0.055 μg/ml (n =108), borderline if 0.045 ≤ HpSAg < 0.055 μg/ml (n = 10) and negative if HpSAg < 0.045 μg/ml (n = 73).

It should be noted that the *H.pylori* positive patients had no sever gastrointestinal complications and did not require to urgent classic treatment for the infection. Twenty-two patients with positive *H.pylori* test did not participants in treatment and the rest were assigned randomly into 3 groups: A) STT: Standard triple therapy including omeprazole 20 mg, clarithromycin 500 mg, amoxicillin 1000 mg, twice a day for 14 days (n = 33); B) BSP: 6 g/d broccoli sprouts powder for 28 days (n = 28); C) STT + BSP: standard triple therapy for 14 days plus 6 g/d broccoli sprouts powder for 28 days (n = 25). Stratified randomization was performed using sealed envelopes for group allocation. Broccoli sprouts powder contain at least 22.5 μmol standardized for sulforaphane per each gram, was purchased from the Cyvex Nutrition Company (CA, USA). To evaluate compliance to intervention and to enquire regarding any possible side effects we contacted participants weekly. One month after treatment, stool samples were obtained again to assess *H.pylori* eradication rates. After the intervention, all the patients in the BSP groups who remained *H.pylori* positive received standard triple therapy.

### Demographics, anthropometrics and clinical measurement

Data on age, educational level, medical history and medications, duration of diabetes and oral anti-diabetes drugs, were collected at baseline by trained interviewers using pretested questionnaires.

Anthropometric indices were measured at baseline and one month after treatment, by trained staff. Weight was measured to the nearest 100 g with minimum cloths, without shoes, using digital scale. Height was measured to the nearest 0.5 cm, using a tape meter while the participants were in a standing position without shoes. Waist circumference (WC) were measured to the nearest 0.1 cm (at anatomical landmarks), at the widest portion, without any pressure to the body using a non-stretched tape meter. Body mass index (BMI) was calculated as weight (kg) divided by square of the height (m^2^).

Blood pressure (BP) measured twice, on the right arm, after a 15-minute rest in the sitting position, using a standardized mercury sphygmomanometer. The mean of the two measurements was considered as the participant's BP.

### Biochemical measurement

Venous blood samples were obtained after 12-h fasting at baseline and 1-month after treatment and stored at the laboratory of Research Institute for Endocrine Science until assay. Fasting serum glucose, total cholesterol and triglycerides was measured by the enzymatic colorimetric method using kits (Pars Azmoon Company, Tehran, Iran). High density lipoprotein cholesterol was measured after precipitation of apo B containing lipoproteins with phosphotungstic acid using kit (Pars Azmoon Company, Tehran, Iran). LDL-C was calculated according to the Friedewald equation. Serum hs-CRP (pg/ml) concentration was measured using the enzyme-linked immunosorbent assay (ELISA) kit (Diagnostics Biochem Canada Inc., Ontario, Canada). Inter- and Intra-assay coefficients of variation of all assays were < 5%. Atherogenic lipid and lipoprotein ratio including TG/HDL, TC/HDL and LDL/HDL ratio were calculated at baseline and 1-month after treatment.

### Statistical methods

The sample size was designed to detect a 0.05 μg/ml difference among groups in *H.pylori* stool antigen with 95% CI and 90% power, and with regard to the possible loss of samples was calculated for 25 patients in each group. All statistical analyses were conducted on the intention-to-treat principle and included all negative HpSAg participants at the follow-up. The Kolmogorov-Smirnov test was used to test for a normal distribution. To compare the means between the three groups at baseline one-way ANOVA or Kruskal-Wallis test were used. To compare baseline and after treatment-values in each group paired t-test or Mann–Whitney test were used. The general linear model (ANCOVA) was used to compare means of the variables after treatment and obtain the main effect of each regimen (STT, BSP, STT + BSP). When the analysis indicated a significant effect of treatment, the groups were compared pair-wise by the Bonferroni. All Statistical analyses were performed using SPSS (version 16.0; SPSS, Inc., Chicago, IL, USA). A *P* value < 0.05 was considered significant.

## Results

**Figure 1:**
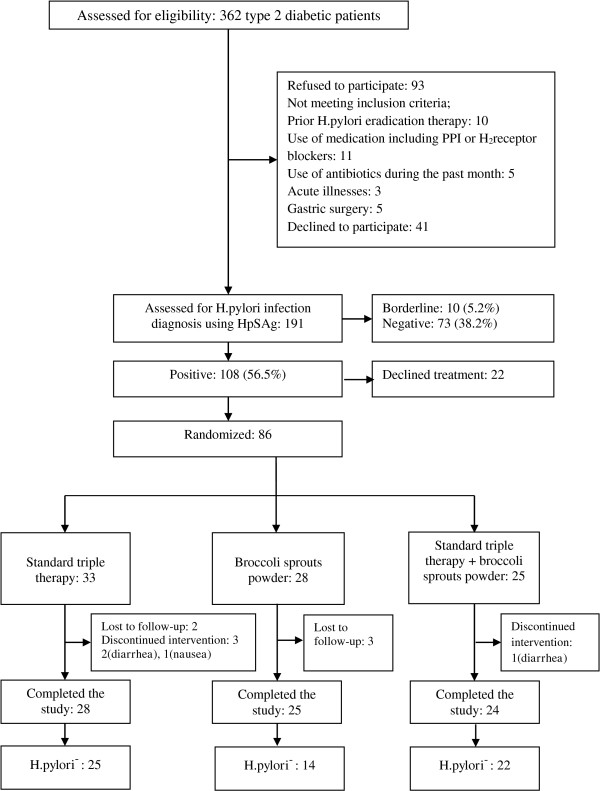
Flow chart of the study.

Of eighty-six randomized patients, seventy-seven completed the study [STT (n = 28), BSP (n = 25), STT + BSP (n = 24)] Figure 1; the mean age, weight and waist circumference of participants was 51 ± 13 years, 81 ± 14 kg and 100 ± 11 cm, respectively. Forty-five percent of the participants were men. Sixty-eight (88.3%) patients with type 2 diabetes were treated with oral anti-diabetes, 2 (2.5%) with a combination of insulin and oral anti-diabetics and 7 (9.1%) by diet per se. There were no significant differences between the three groups in medications, including anti-diabetes drugs, lipid lowering or antihypertensive drugs.

Mean fasting glucose levels at baseline were 164 ± 59, 154 ± 46, and 157 ± 51 mg/dl in groups A, B and C, respectively. Mean HpSAg levels at baseline were similar in the three groups (0.29 ± 0.25, 0.24 ± 0.20, and 0.25 ± 0.23 in the group A, B and C, respectively). No significant differences between the groups were observed at baseline examination for age, weight, waist circumference, systolic and diastolic blood pressure, duration of diabetes, and biochemical measurements. The most common adverse events observed during the intervention were diarrhea (9.6%) and nausea (3.8%) in patients who received standard triple therapy.

The eradication rates of *H.pylori*, assessed by HpSAg, were 89.3%, 56.0%, and 91.7% in the STT, BSP, and STT + BSP groups, respectively. Final analyses were conducted on all participants with negative HpSAg at the follow-up. Baseline and post-treatment anthropometrics as well as cardiovascular risk factors in patients with successfully eradicated *H.pylori* in the three groups are compared in Table 1. After the treatment, both systolic and diastolic blood pressure significantly decreased in patients who were treated with standard triple therapy plus broccoli sprouts powder (*P < 0.05*). Serum total cholesterol moderately but non-significantly increased in the STT and STT + BSP groups, and remained unchanged in the BSP group. As compared to baseline, serum triglycerides significantly increased in the STT group (154 ± 109 *vs.* 131 ± 80, *P < 0.05*); triglycerides to HDL-C ratio, a main atherogenic index of plasma, also increased in patients treated with STT per se (4.5 ± 2.9 at baseline *vs.* 5.5 ± 3.9 after the treatment, *P < 0.05*); no significant changes in LDL-C and HDL-C levels of the 3 groups were observed. Serum hs-CRP levels after the treatment significantly decreased in patients treated with BSP per se (3.0 ± 2.5 at baseline *vs.* 2.3 ± 2.1 after the treatment, *P < 0.05*), and remained unchanged in other groups. When, after treatment values adjusted for baseline levels were compared between the three groups, triglyceride levels were significantly higher in STT group as compared with others, and treatment effects of STT on increased levels of triglycerides was significant (*P for treatment effect <0.05*). The TG/HDL-C ratio in the three groups ranked as follows: STT > STT + BSP > BSP; differences however not statistically significant. Serum level of hs-CRP in the BSP group was non-significantly lower than the STT + BSP and STT groups, respectively Table 2.

**Table 1:** **Baseline and after treatment values in successful *H.pylori *** **eradicated patients in the three groups**

	**Standard triple therapy *(n = 25)***		**Broccoli sprouts powder *(n = 14)***		**Standard triple therapy + Broccoli sprouts powder *(n = 22)***	
	**Baseline**	**After treatment**	**Baseline**	**After treatment**	**Baseline**	**After treatment**
Weight *(kg)*	83.3 ± 15.8	82.5 ± 16.4	75.7 ± 16.1	75.3 ± 15.8	73.8 ± 10.7	72.8 ± 10.5
Waist circumference *(cm)*	104 ± 10.7	102 ± 13.2	94.8 ± 14.7	95.5 ± 12.4	94.1 ± 8.3	92.6 ± 8.6
Systolic blood pressure *(mmHg)*	130 ± 20	125 ± 11	125 ± 20	119 ± 24	136 ± 23	122 ± 14^a^
Diastolic blood pressure *(mmHg)*	80.4 ± 16.2	79.4 ± 8.6	80.4 ± 12.4	76.8 ± 16.8	89.8 ± 16.2	80.4 ± 10.4^a^
Total cholesterol *(mg/dl)*	158 ± 38	166 ± 40	157 ± 32	156 ± 11	152 ± 31	165 ± 33
Triglycerides *(mg/dl)*	131 ± 80	154 ± 109^a^	107 ± 50	110 ± 51	113 ± 88	114 ± 55
HDL-cholesterol *(mg/dl)*	30.1 ± 4.0	29.0 ± 3.4	29.3 ± 2.6	28.7 ± 2.1	30.1 ± 3.6	29.1 ± 2.6
LDL-cholesterol *(mg/dl)*	104 ± 29	108 ± 30	111 ± 27	108 ± 31	106 ± 28	114 ± 26
Triglycerides/HDL-C	4.5 ± 2.9	5.5 ± 3.9^a^	3.8 ± 1.9	3.9 ± 1.9	3.9 ± 2.7	4.0 ± 2.1
Total cholesterol/HDL-C	5.6 ± 1.6	5.8 ± 1.6	5.5 ± 1.5	5.4 ± 1.5	5.6 ± 1.4	5.7 ± 1.3
LDL-C/HDL-C	3.5 ± 0.9	3.8 ± 1.1	3.8 ± 1.2	3.8 ± 1.1	3.6 ± 1.2	4.0 ± 1.1
hs-CRP *(mg/l)*	3.3 ± 2.5	3.1 ± 2.4	3.0 ± 2.5	2.3 ± 2.1^a^	2.4 ± 1.9	2.5 ± 2.3

All values are mean ± SD.

There was no significant difference in the clinical variables between the three groups (analysis of variance was used).

^a^Significantly different from baseline values (student paired t-test was used) *p < 0.05.*

**Table 2:** **Cardiovascular risk factors after treatment in successful *H.pylori *** **eradicated patients in the three groups**

	**Standard triple therapy *(n = 25)***	**Broccoli sprouts powder *(n = 14)***	**Standard triple therapy + Broccoli sprouts powder *(n = 22)***	***p *** **for treatment effect**^**a**^
Systolic blood pressure *(mmHg)*	126 ± 4	122 ± 5	120 ± 4	0.63
Diastolic blood pressure *(mmHg)*	81 ± 3	78 ± 3	77 ± 3	0.68
Total cholesterol *(mg/dl)*	166 ± 8	156 ± 13	166 ± 8	0.82
Triglycerides *(mg/dl)*	154 ± 14	99 ± 29^b^	118 ± 15^b^	0.05
HDL-cholesterol *(mg/dl)*	29.0 ± 0.6	28.7 ± 1.1	29.0 ± 0.7	0.96
LDL-cholesterol *(mg/dl)*	108 ± 6	108 ± 10	114 ± 7	0.76
Triglycerides/HDL-C	5.5 ± 0.6	3.4 ± 1.1	4.2 ± 0.7	0.08
Total cholesterol/HDL-C	5.8 ± 0.3	5.5 ± 0.5	5.7 ± 0.3	0.88
LDL-C/HDL-C	3.8 ± 0.2	3.8 ± 0.4	4.0 ± 0.2	0.75
hs-CRP *(mg/l)*	2.9 ± 0.5	2.3 ± 0.8	2.7 ± 0.5	0.08

All values are mean (estimated marginal means based on baseline values as covariate in general linear model) ± SE.

^a^Calculated by using a general linear model with after treatment values as dependent variables, baseline values as covariates and treatment group as a fixed factor.

^b^Significantly different from STT groups (Bonferroni pairwise comparison was used; *P < 0.05*).

## Discussion

The results of this clinical trial showed that compared to the common standard triple therapy, broccoli sprouts powder in addition to its considerable effect on *H.pylori* eradication, could also have favorable effects on cardiovascular risk factors following the treatment.

The possible synergistic hypotensive effect of broccoli sprouts powder and standard triple therapy was an important finding observed following *H.pylori* eradication in this study; a moderate decrease of systolic and diastolic blood pressure was however observed in all patients with successfully eradicated *H.pylori*, this decrease was as follows BSP + STT > BSP > STT, and was only significant in BSP + STT treatment group. The association between *H.pylori* infection and elevated blood pressure is controversial [13,14], but it seems that H.pylori eradication could improve blood pressure especially in hypertensive patients [15]. The hypotensive effect of broccoli sprouts is also controversial; supplementation of spontaneously hypertensive stroke-prone rat with broccoli sprouts significantly decreased blood pressure [16], while ingestion of broccoli sprouts had not significant effect on blood pressure and endothelial function measured by flow mediated dilation in hypertensive patients [17]. The antioxidative and anti-inflammatory property of sulforaphane in broccoli sprouts have been proposed as a main mechanism contributing to improvement of endothelial function and blood pressure.

The most interesting finding we observed one month after the treatment was a significant increase in serum triglyceride levels in patients with successfully eradicated *H.pylori* who received standard triple therapy; while no similar adverse effect was observed in patients who were treated alternatively with BSP, or received BSP as complementary treatment of standard triple therapy. Serum triglycerides have been identified as major contributing factor and independent predictor of coronary heart disease in patients with type 2 diabetes, and lowering triglyceride levels has always considered a main clinical target in these patients [18]; it has been suggested that even a temporary and short-term increase in triglyceride levels could be accompanied by development of insulin resistance, endothelial damage and dysfunction, especially in patients with diabetes, via increased levels of intracellular adhesion molecule-1, vascular cell adhesion molecule-1, and E-selectin, as well as oxidative stress [19-21]. Another important risk factor outcome observed following the *H.pylori* eradication was clinically considerable, but not statistically significant, elevated triglyceride to HDL-C ratio in the STT group; TG/HDL-C ratio is a main atherogenic lipid parameter that is directly related to lipoprotein particle size and especially small LDL-dense particles in the plasma [22,23]; patients with type 2 diabetes with high TG/HDL-C ratio are potentially at a higher risk for increased arterial stiffness and atherosclerosis [24].

However the reason for the undesirable effects observed of standard triple therapy following *H.pylori* eradication in patients with type 2 diabetes is not clear, but the beneficial effects of broccoli sprouts supplementation in improvement of lipids and lipoproteins are well explained. The potential effects of broccoli sprouts and its bioactive components including isothiocyanate sulforaphane on lipid and lipoprotein metabolism have previously been investigated; phytonutrient compounds in broccoli sprouts can bind with bile acids and reduce fat absorption, inhibit lipoprotein lipase activity in adipose tissue, decrease gene expression and the activity of key lipogenic enzymes, including diacylglycerol acyltransferases, fatty acid synthase, and acyl-CoA-cholesterol acyltransferase; moreover, indole glucosinolates in broccoli sprouts reduce apolipoprotein B secretion, which is a primary apolipoprotein of low-density lipoproteins [25-27]. Human studies have also confirmed the hypolipidemic effects of broccoli sprouts [28,29].

Increased serum levels of hs-CRP are considered as a marker of *H.pylori*-induced inflammation in gastric mucosa [30,31]; moreover sub-clinical inflammation and increased level of hs-CRP have been identified as a main contributor to development of insulin resistance and diabetes complications, and also an independent risk factor and powerful predictor of incident cardiovascular events [32-34]. Current data are controversial in relation to *H.pylori* eradication and improvement of inflammatory parameters [35-38]. In our study, a significant decrease in serum hs-CRP levels was observed only in patients with successfully eradicated *H.pylori* treated with broccoli sprouts powder per se, while hs-CRP levels remained unchanged in the other two groups. The anti-inflammatory properties of broccoli sprouts are mainly attributed to sulforaphane which could inhibit cytokine production through the activation of nuclear factor (erythroid-derived 2)-like-2 pathway and induction of NAD(P)H:quinon oxidoreductase 1, an antioxidant phase II protein [39]; SFN also inhibits production of inflammatory mediators and cytokines including tumor necrosis factor-α, interlukine-6, interlukine-1β, and prostaglandins through inhibition of nuclear factor-κB transcriptional activity, as a key modulator of pro-inflammatory processes [40,41].

To the best of our knowledge, this clinical trial was the first study investigating comparative effects of broccoli sprouts powder and standard triple therapy on *H.pylori* infection and cardiovascular risk factor outcomes following the *H.pylori* eradication in patients with type 2 diabetes. The dose and duration of BSP treatment used in this study were limited and therefore determination of optimum dose and duration were not possible. Last, but not least, our study was not adequately powered to explore possible differences between groups in term of eradication rate. Moreover, as expected because of a small sample size our power was inadequate for subgroup analysis. Further studies on the current topic are therefore recommended.

In conclusion, the study showed that standard triple therapy could increase serum triglycerides and TG/HDL-C ratio, as main cardiovascular risk factors, following *H.pylori* eradication in patients with type 2 diabetes; while broccoli sprouts powder, in addition to considerable effect on *H.pylori* eradication and related inflammation, could reduce the unfavorable effects of STT. Returning to the hypothesis posted at the beginning of the study, it is now possible to state that potential undesirable outcomes, including cardiovascular risk factors especially in patients with type 2 diabetes, during common medications used for *H.pylori* eradication should be considered. Moreover, use of natural antibacterial agents, including nutraceuticals and functional ingredients such as broccoli sprouts, beyond improving the efficacy of common treatment of *H.pylori* infection, may attenuate undesirable side effects, and consequently lead to health promoting outcomes in the patients.

## Competing interests

The authors declare that they have no competing interests.

## Authors’ contribution

The project was designed and implemented by ZB, PM and HZ Data were analyzed and interpreted ZB and MG ZB, MG, FA prepared the manuscript. PM, and FA supervised overall project. All authors read and approved the final version of manuscript.
